# Patterns of Peripheral Blood B-Cell Subtypes Are Associated With Treatment Response in Patients Treated With Immune Checkpoint Inhibitors: A Prospective Longitudinal Pan-Cancer Study

**DOI:** 10.3389/fimmu.2022.840207

**Published:** 2022-04-01

**Authors:** Dominik A. Barth, Stefanie Stanzer, Jasmin A. Spiegelberg, Thomas Bauernhofer, Gudrun Absenger, Joanna Szkandera, Armin Gerger, Maria A. Smolle, Georg C. Hutterer, Sascha A. Ahyai, Tobias Madl, Florian Posch, Jakob M. Riedl, Christiane Klec, Philipp J. Jost, Julia Kargl, Martin H. Stradner, Martin Pichler

**Affiliations:** ^1^Division of Oncology, Department of Internal Medicine, Medical University of Graz, Graz, Austria; ^2^Department of Orthopaedics and Trauma, Medical University of Graz, Graz, Austria; ^3^Department of Urology, Medical University of Graz, Graz, Austria; ^4^Gottfried Schatz Research Center, Molecular Biology and Biochemistry, Medical University of Graz, Graz, Austria; ^5^BioTechMed-Graz, Graz, Austria; ^6^Division of Hematology, Department of Internal Medicine, Medical University of Graz, Graz, Austria; ^7^Otto Loewi Research Center, Division of Pharmacology, Medical University of Graz, Graz, Austria; ^8^Division of Rheumatology and Immunology, Department of Internal Medicine, Medical University of Graz, Graz, Austria; ^9^Department of Experimental Therapeutics, The University of Texas MD Anderson Cancer Center, Houston, TX, United States

**Keywords:** B cells, cancer, immune checkpoint inhibitor therapy, lymphocytes, response

## Abstract

**Background:**

Immune checkpoint inhibitors (ICIs) have revolutionized systemic anti-tumor treatments across different types of cancer. Nevertheless, predictive biomarkers regarding treatment response are not routinely established yet. Apart from T-lymphocytes, the humoral immunity of B-lymphocytes is studied to a substantially lesser extent in the respective setting. Thus, the aim of this study was to evaluate peripheral blood B-cell subtypes as potential predictors of ICI treatment response.

**Methods:**

Thirty-nine cancer patients receiving ICI therapy were included into this prospective single-center cohort study. All had a first blood draw at the date before treatment initiation and a second at the time of first response evaluation (after 8-12 weeks). Seven different B-cell subtypes were quantified by fluorescence-activated cell sorting (FACS). Disease control- (DCR) and objective response rate (ORR) were co-primary study endpoints.

**Results:**

Overall, DCR was 48.7% and ORR was 25.6%, respectively. At baseline, there was no significant association of any B-cell subtype with neither DCR nor ORR. At the first response evaluation, an increase in the frequency of CD21^-^ B-cells was a statistically significant negative predictor of response, both regarding DCR (OR=0.05, 95%CI=0.00-0.67, *p*=0.024) and ORR (OR=0.09, 95%CI=0.01-0.96, *p*=0.046). An increase of the frequency of switched memory B-cells was significantly associated with reduced odds for DCR (OR=0.06, 95%CI=0.01-0.70, *p*=0.025). Patients with an increased frequency of naïve B-cells were more likely to benefit from ICI therapy as indicated by an improved DCR (OR=12.31, 95%CI=1.13-134.22, *p*=0.039).

**Conclusion:**

In this study, certain B-cell subpopulations were associated with ICI treatment response in various human cancer types.

## 1 Introduction

The recent introduction of immune checkpoint inhibitors (ICIs) as systemic treatment modality of solid malignancies has significantly improved patients’ survival outcomes across a variety of human cancer types (1). By monoclonal antibody-mediated blocking of certain IC-molecules, mainly the programmed cell death protein 1 (PD-1), programmed cell death ligand 1 (PD-L1), and cytotoxic T-lymphocyte-associated protein 4 (CTLA4), cancer cells immune evasion can be reprogrammed and an immune response against cancer cells can be induced (2).

Although a substantial number of patients respond with long-lasting remissions or even complete remissions (CRs) upon ICI treatment initiation, a significant proportion of patients does not respond to ICI treatment at all (2). Depending on cancer entity, predictive biomarkers, such as the PD-(L)1 expression and related scores are routinely used as a potential decision tool and as predictive biomarkers in clinical practice in certain types of cancer (3, 4), yet their predictive ability is nevertheless inaccurate in most treatment settings. Besides sometimes complicated and dangerous side effects, these novel agents carry substantial treatment costs, which warrant the ongoing search for predictive biomarkers all the more, to better select appropriate candidates for ICI therapy (5–7).

To date, the crucial role of T-lymphocytes in the mode of action of ICIs in cancer therapy is well known (8–12). Nonetheless, recent evidence suggests a major role of B-lymphocytes and various B-cell subtypes in ICI-based cancer treatment (13). In addition, B-cells forming tertiary lymphoid structures (TLS) in the tumor microenvironment may also play an important role for an efficient anti-cancer immune response upon ICI therapy (14). However, a better functional understanding of different B-cell populations in ICI treatment, as well as prospective clinical studies evaluating B-cells as potential biomarkers for treatment efficacy in cancer patients undergoing ICI therapy are in general scarce. For instance, in cancer the relevance of CD21^-/low^ B-cells, a subset which linked to several autoimmune diseases (15) and are considered an exhausted and anergic B-cell subpopulation (15–19), undefined yet. Recently, the frequency of peripheral blood B-cells was associated with response rates in a retrospective cohort study, albeit an analysis of the B-cell subtypes involved was missing (20). As such, the potential value of peripheral blood B-cells as an easily accessible blood-based biomarker for the prediction of treatment response currently remains undefined. The aim of this study was to prospectively evaluate the potential predictive value of peripheral blood B-cells for ICI treatment efficacy and to monitor the change of B-cell population levels during ICI treatment in a human pan-cancer setting.

## 2 Methods

Forty-five consecutive cancer patients who were treated at the Division of Oncology, Department of Internal Medicine, Medical University of Graz and received ICI therapy between January 2017 and December 2020, were included into this prospective longitudinal biomarker study. Patients of all cancer types who were older than 18 years of age and who received mono- or combination ICI were included in the study. Patients with preexisting autoimmune diseases were excluded from the study.

All patients underwent a first blood draw at the date before treatment initiation and a second one 8-12 weeks after initiation of ICI treatment at the time of the first response evaluation. All patients were seen by an experienced oncologist before each treatment administration and they were evaluated for treatment response every 8-12 weeks by CT- or MRI-scans as appropriate, considering RECIST version 1.1 criteria. All blood samples were delivered in a standardized manner after collection to the Laboratory of the Division of Rheumatology and Immunology, Department of Internal Medicine, Medical University of Graz. All assays were calibrated and standardized for routine clinical sample testing.

The absolute number of CD19^+^ B cells was determined by flow cytometry as previously described (21). B-cell subsets were analyzed in peripheral blood, mononuclear cells isolated from lithium heparin blood by Ficoll gradient density centrifugation. One million cells were incubated with antibodies against CD19, IgD, CD24, CD38, CD27, CD86, CD21, and IgM (Miltenyi Biotec, Bergisch Gladbach, Germany) (**Supplementary Table 1**). Samples were measured using a FACS Canto II flow cytometer (BD Biosciences, Franklin Lakes, NJ, USA). Data were analyzed using the FACSDiva software (BD Biosciences) using a gating strategy as published previously (22) (**Supplementary Figure 1**).

B-cell subsets were classified according to the EUROclass gating strategy by Wehr et al. (23) as follows: CD21^-^ (CD19^+^ CD38^-^ CD21^-^), unswitched memory B cells (or marginal zone) B-cells (CD19^+^ CD27^+^ IgD^+^ IgM^+^), naïve B-cells (CD19^+^ CD27^-^ IgD^+^ IgM^+^), transitional B-cells (CD19^+^ CD38^++^ IgM^++^), switched memory B-cells (CD19^+^ CD27^+^ IgD^-^ IgM^-^), regulatory B-cells (CD19^+^ CD24^+^ CD38^++^) and plasmablasts (CD19^+^ CD38^++^ IgM^-^). Regulatory B-cells were defined as proposed by Das et al. (24) and Blair et al. (25).

### 2.1 Statistical Analysis

Co-primary endpoints of this study were disease control rate (DRC), defined as the rate of patients who experienced CR, partial remission (PR) or stable disease (SD), and objective response rate (ORR), defined as the rate of patients who experienced CR or PR.

To assess for the association of clinico-pathological parameters with the B-cell measurements, the Mann-Whitney-U-test and the Kruskal-Wallis-test were used where appropriate. At baseline, uni- and multivariable logistic regression models were performed to assess whether different B-cell subpopulations might predict for treatment responses, whereby odds ratios (ORs) and 95% confidence intervals (CIs) are reported. Throughout the analysis, ORs for different B-cell subtypes were calculated and reported as per 1000, 100 or 10 units increase, as appropriate. Due to the hypothesis-generating character of our study, multivariable analyses were only adjusted for tumor type. To avoid perfect prediction of the outcome in the multivariable logistic regression analysis at the second blood draw, tumor types for adjustment were summarized as Non-Small Cell Lung Cancer (NSCLC), genitourinary [Renal cell carcinoma (RCC) and urothelial carcinoma (UC)], gastrointestinal (CRC, gastric and cholangiocellular carcinoma) and head and neck cancer. Increase vs. decrease or no change, absolute change (2^nd^ blood draw – 1^st^ blood draw) and relative change [(2^nd^ blood draw - 1^st^ blood draw)/1^st^ blood draw*100] were considered in the analysis of changes in B-cell levels after 8-12 weeks of ICI treatment. Changes of B-cell levels were assessed using the Wilcoxon-Sign-Rank Test. A two-sided *p*-value of <0.05 was considered statistically significant in all analyses.

All statistical analyses were performed using Stata for Windows Version 16.1 (StataCorp LP, Collage Station, TX, USA). Box plots were created using GraphPad Prism Version 9.1.2 for Windows (GraphPad Software, San Diego, California, USA).

### 2.2 Ethics

Written informed consent was obtained from each patient included into this study. This study was approved by the local ethics committee of the Medical University of Graz (29-593 ex 16/17).

## 3 Results

Overall, 45 patients treated with ICIs were included into this prospective single-center cohort study. One patient was excluded due to loss of follow-up. Five patients did not have B-cell FACS at treatment initiation and were subsequently excluded from analyses. Thus, 39 patients were included into the final analysis.

### 3.1 Baseline Characteristics

Twenty-seven (69%) patients were male, and the median age was 64 years. Fourteen (36%) patients had histologically verified NSCLC, 9 (23%) patients had RCC, 7 (18%) patients had UC of the urinary bladder, 4 (10%) patients had head and neck squamous cell carcinoma and one (3%) patient each had gastric or cholangiocellular carcinoma. More than half of the patients (51%) were treated either with pembrolizumab (51%) or nivolumab (43%), whereas one patient each received treatment with atezolizumab or a combination therapy of nivolumab plus ipilimumab. More than half (51%) of the study population were in a 2^nd^ line treatment setting, 14 (36%) patients received 1^st^ line treatment and 5 (13%) patients received immunotherapy as a 3^rd^ line treatment (**Supplementary Table 2**).

PR was achieved in 10 (26%) patients. No patient experienced CR during ICI treatment. Nine (23%) patients had SD and 20 (51%) patients had progressive disease (PD) at the time of first response evaluation after 8-12 weeks of ICI treatment. In the overall cohort, DCR was 48.7% and ORR was 25.6%. Due to the limited sample size and the hypothesis-generating character of the study, multivariable analyses were only adjusted to tumor type in all further analysis. However, the clinico-pathological parameters including age, sex, tumor type, treatment line, treatment modality, and histology were neither associated with DCR nor ORR in the univariable logistic regression model (all *p*>0.05, data not shown).

### 3.2 Association of Response-Rates With B-Cell Levels at Baseline

Summary measures of the B-cell subpopulations at baseline and respective associations with clinico-pathological parameters are shown in **Table 1**. Interestingly, absolute B-cell counts were significantly higher in female (median 6826 cells/µl; IQR 1369-10,018 cells/µl) than male patients (median 3053 cells/µl; IQR 639-5032 cells/µl) [*p*=0.0452] and the B-cell frequency was significantly higher in smokers (median 3.25%) vs. never-smokers (median 1.80%) [p=0.0119]. Apart from this observation, no differences in the B-cell distribution depending on clinico-pathological parameters were recorded.

**Table 1:** Association of B-cell subpopulations with clinico-pathological parameters.

			*p*-values				
		Median [IQR]	Age (>65yrs.)	Gender	Smoker	Tumor type	Treatment line
**Lymphocytes**	absolute count	145,922 [93,985 - 190,337]	0.6642	0.3745	0.8786	0.3338	0.5102
**B-cells - total**	absolute count	3725 [928 – 6,726]	0.4746	**0.0452**	0.0612	0.1768	0.6281
	% lymphocytes	2 [1.19 – 5.33]	0.4619	0.7348	**0.0119**	0.1249	0.5249
**CD21-negative**	absolute count	860 [246 – 2149]	1.0000	0.2098	0.0696	0.1477	0.7930
	% B cells	27 [21.4 – 48.4]	0.0642	0.4044	0.4234	0.5512	0.3513
**unswitched memory B-cells**	absolute count	241 [56 – 367]	0.8615	0.4048	0.1640	0.4200	0.8512
	% B cells	6.45 [4.31 – 9.18]	0.6043	0.1782	0.4780	0.3154	0.3909
**Transitional Zone B-cells**	absolute count	45 [12 – 193]	0.4876	0.9461	0.4735	0.3978	0.3483
	% B cells	1.55 [0.64 – 4.95]	0.6091	0.5732	0.8951	0.6548	0.1580
**Naive B-cells**	absolute count	1,818 [590 – 4,095]	0.3766	0.0606	0.2138	0.4098	0.6164
	% B cells	59.2 [48.1 – 78.3]	0.2429	0.3745	0.7919	0.5709	0.0664
**Switched memory B-cells**	absolute count	419 [179 – 915]	0.7421	0.0926	0.0934	0.2321	0.6357
	% B cells	16.7 [8.26 – 24.2]	0.5704	0.7584	0.8950	0.4727	0.1916
**CD24^+^CD38^++^ Regulatory B-cells**	absolute count	0 [0 – 0] Maximum 2	0.5198	1.0000	0.3521	0.1714	0.8889
	% B cells	0 [0 – 0] Maximum 0.33	0.4532	0.8743	0.1715	0.1329	0.9366
**Plasmablasts**	absolute count	78 [19 – 266]	0.9275	0.0844	0.4737	0.2484	0.7873
	% B cells	2.97 [1.48 – 5.49]	0.3615	0.2578	0.5499	0.6135	0.9364

IQR, interquartile range; significant values are highlighted in bold.

**Figure 1:**
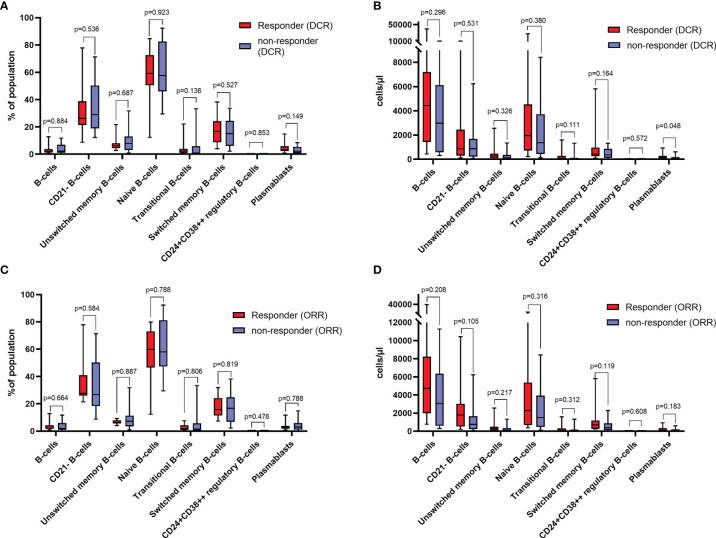
**(A)** Relative distribution of different B-cell subtypes in responders and non-responders as indicated by disease control rate (DCR) at baseline. **(B)** absolute counts of different B-cell subtypes in responders and non-responders as indicated by DCR at baseline. **(C)** Relative distribution of different B-cell subtypes in responders and non-responders as indicated by objective response rate (ORR) at baseline. **(D)** absolute counts of different B-cell subtypes in responders and non-responders as indicated by ORR at baseline. Values in **(A, C)** are percentages of total lymphocytes (for B-cells), and percentages of total B-cells (for B-cell subsets). All groups compared by Mann-Whitney-U-test.

Except for plasmablasts, which were significantly increased in responders, as indicated by DCR (*p*=0.048), there was no difference in the distribution of B-cell subtypes in responders and non-responders, as indicated by DCR and ORR (**Figures 1** **A–D**).

At baseline, there was no significant association, neither with DCR nor ORR of total B-cell, CD21^-^ B-cells, unswitched memory B-cells, transitional zone B-cells, naïve B-cells, switched memory B-cells, CD24^+^CD38^++^ regulatory B-cells and plasmablasts measurements in both, univariable logistic regression models, as well as in the multivariable analysis adjusted for tumor entity (**Table 2**).

**Table 2:** B-cell measurements and associations with disease control rate (DCR) and objective response rate (ORR) at baseline.

		Disease Control Rate				Objective Response Rate			
Variable		Univariable Analysis		Multivariable Analysis		Univariable Analysis		Multivariable Analysis	
		OR (95%CI)	*p*-value	OR (95%CI)	*p*-value	OR (95%CI)	*p*-value	OR (95%CI)	*p*-value
**Lymphocytes**	count*	1.01 (0.99-1.02)	0.067	1.01 (1.00-1-02)	**0.037**	1.01 (1.00-1.02)	**0.039**	1.01 (1.00-1.02)	**0.045**
**B-cells – total**	count*	1.08 (0.93-1.26)	0.300	1.15 (0.92-1.43)	0.222	1.10 (0.95-1.28)	0.189	1.10 (0.95-1.29)	0.200
	% lymph	0.96 (0.79-1-17)	0.679	0.99 (0.80-1-23)	0.925	1.02 (0.80-1-31)	0.853	1.00 (0.80-1.25)	0.984
**CD21- B-cells**	count*	1.13 (0.81-1.59)	0.462	1.19 (0.81-1-76)	0.374	1.34 (093-1.96)	0.117	1.36 (0.92-2.02)	0.124
	% B***	0.87 (0.60-1.25)	0.441	0.86 (0.58-1.28)	0.463	1.05 (0.70-1.58)	0.803	1.05 (0.70-1.60)	0.793
**Unswitched memory B-cells**	count**	1.06 (0.92-1-22)	0.445	1.06 (0.91-1.23)	0.462	1.08 (0.94-1.24)	0.254	1.08 (0.94-1.24)	0.260
	% B	0.95 (0.85-1.05)	0.300	0.92 (0.83-1.03)	0.174	0.94 (0.81-1.08)	0.359	0.92 (0.78-1.08)	0.317
**Transitional Zone B-cells**	count**	1.05 (0.88-1.26)	0.580	1.06 (0.87-1.29)	0.539	1.07 (0.89-1.28)	0.459	1.07 (0.89-1.29)	0.446
	% B	1.00 (0.91-1.11)	0.945	0.99 (0.99-1.1)	0.916	0.94 (0.78-1.11)	0.448	0.94 (0.79-1.12)	0.456
**Naive B-cells**	count*	1.08 (0.91-1.28)	0.372	1.11 (0.90-1.39)	0.333	1.11 (0.94-1.30)	0.217	1.11 (0.94-1.30)	0.218
	% B***	0.92 (0.65-1.30)	0.639	0.92 (0.64-1.32)	0.662	0.89 (0.60-1.32)	0.569	0.90 (0.60-1.35)	0.625
**Switched memory B-cells**	count**	1.08 (0.96-1.23)	0.210	1.14 (0.96-1.35)	0.139	1.08 (0.97-1.20)	0.161	1.07 (0.97-1-19)	0.177
	% B***	1.27 (0.65-2.49)	0.489	1.21 (0.60-2.45)	0.600	1.07 (0.50-2.30)	0.849	1.03 (0.46-2.31)	0.942
**CD24^+^CD38^++^ Regulatory B-cells**	count	1.72 (0.42-7.13)	0.453	1.50 (0.34-6.72)	0.596	0.43 (0.05-3.41)	0.422	0.37 (0.04-3.26)	0.370
	% B	NA		NA		NA		NA	
**Plasmablasts**	count**	1.23 (0.87-1.73)	0.245	1.41 (0.89-2.24)	0.146	1.26 (0.90-1.76)	0.174	1.29 (0.91-1.85)	0.157
	% B	1.18 (0.94-1.48)	0.148	1.20 (0.94-1.52)	0.148	0.96 80.76-1.21)	0.731	0.96 (0.76-1.23)	0.756

*per 1000 Unit increase, **per 100 Unit increase, ***per 10 Unit increase; % lymph – percent of total lymphocytes; % B – percent of total B-cells; NA – not applicable, significant values are highlighted in bold.

### 3.3 Change of B-Cell Counts and Frequency During ICI-Treatment

Twenty-seven (69.2%) patients had a second follow-up blood draw at the time point of first response evaluation after the start of ICI treatment, while 12 (30.8%) patients either dropped out of the study before having a second blood draw due to PD, death, unfitness for further treatment, or they had received no ICI therapy within 8 weeks for any other reason.

**Figure 2:**
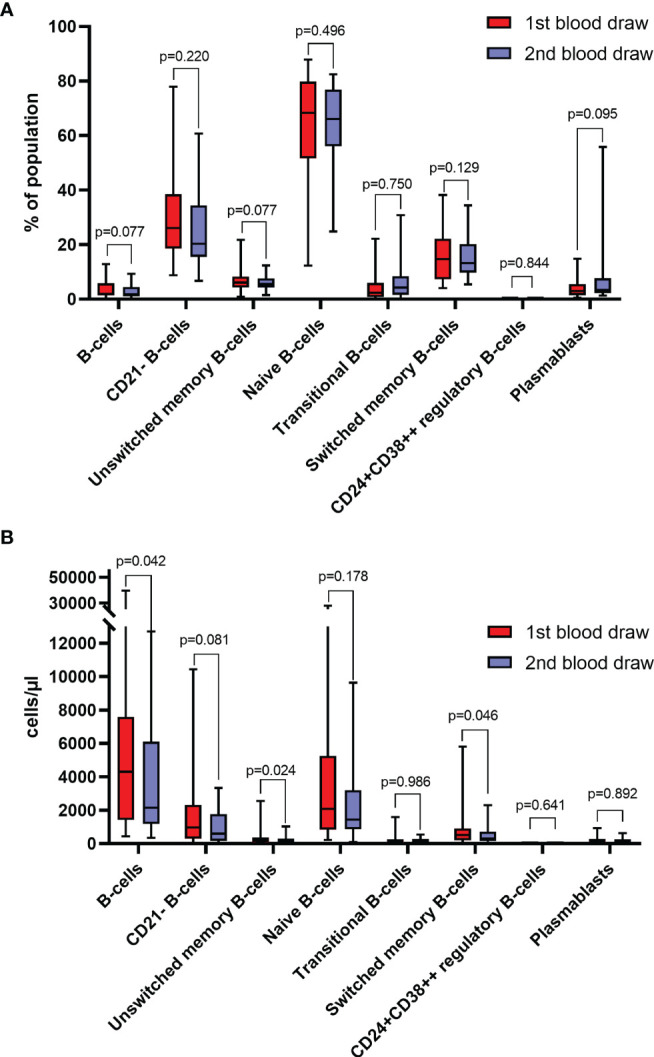
**(A)** Frequencies of different B-cell subtypes at baseline (1^st^ blood draw) and after 8-12 weeks (2^nd^ blood draw) of immune checkpoint inhibitor **(ICI)** treatment (n=27). Values are in percentages of total lymphocytes (for B-cells), and percentages of total B-cells (for B-cell subsets). **(B)** Absolute counts of different B-cell subtypes at baseline (1^st^ blood draw) and after 8-12 weeks (2^nd^ blood draw) of ICI treatment (n=27). All groups compared by Mann-Whitney-U-test.

Regarding absolute counts at the first time of response evaluation, 11 (41%) patients had an increase in lymphocytes, 8 (30%) patients had an increase in total B-cells, 10 (37%) patients had an increase in CD21^-^ B-cells, 7 (26%) patients had an increase in unswitched memory B-cells, 10 (37%) patients had an increase in naïve B-cells, 13 (48%) patients had an increase in transitional zone B-cells, 9 (33%) patients had an increase in switched memory B-cells, and 15 (56%) patients had an increase in plasmablast counts. No individual showed an increase in CD24^+^CD38^++^ regulatory B-cell counts. Significant changes in absolute counts of total B-cells (median change -789 cells/µl, *p*=0.0422), unswitched memory B-cells (median change -76 cells/µl, *p*=0.0237) and switched memory B-cells (median change -140 cells/µl, *p*=0.0463) could be observed, which were significantly lower at the time of response evaluation of ICI treatment in the overall cohort. However, there was no significant change of lymphocyte counts (*p*=0.3997), CD21^-^ B-cells (*p*=0.0815), naïve B-cells (*p*=0.1775), transitional zone B-cells (*p*=0.9859), CD24^+^CD38^++^ regulatory B-cells (0.6406), and plasmablasts (*p*=0.8919) (**Figure 2B**).

Regarding the frequency of B-cell subpopulations, 10 (37%) and 12 (44%) patients showed an increase in total B-cells and CD21- B-cells, respectively. Fourteen (52%) patients each had an increase in unswitched memory B-cells, naïve B-cells, or switched memory B-cells, whereas 18 (67%) patients each showed increased frequencies transitional zone B-cells or plasmablasts after ICI treatment. No single patient had an increase in CD24^+^CD38^++^ regulatory B-cell frequency. Yet, no significant changes in the frequencies of total B-cells (*p*=0.0772), CD21^-^ B-cells (*p*=0.2198), unswitched memory B-cells (*p*=0.5149), naïve B-cells (*p*=0.7496), transitional zone B-cells (*p*=0.1286), switched memory B-cells (*p*=0.9247), CD24^+^CD38^++^ regulatory B-cells (*p*=0.6406) and plasmablasts (*p*=0.0954) were observed (**Figure 2A**).

#### 3.3.1 Association of Changes in B-Cells With Response Rates

Patients with an increase in the frequency of naïve B-cells showed significantly greater odds for DCR in both, univariable (OR=7.00, 95%CI=1.10-44.61, *p*=0.039) and multivariable (OR=12.31, 95%CI=1.13-134.22, *p*=0.039) analyses. There was no statistically significant relationship between the frequency of naïve B-cells and treatment response, as indicated by ORR in uni- and multivariable analyses (**Table 3**). Yet, although a higher absolute change was not significantly associated with DCR and ORR in the univariable model, multivariable adjustment for tumor entity revealed a significant relationship of an absolute increase in the frequency of naïve B-cells with DCR (per 10-unit increase: OR=1.94, 95%CI=1.05-3.59, *p*=0.035) and ORR (per 10-unit increase: OR=2.15, 95%CI=1.07-4.34, *p*=0.033) (**Table 4**). There was no significant association with DCR and ORR when considering relative changes of naïve B-cell measurements (**Supplementary Table 3**).

**Table 3:** Increase vs. decrease or no change (reference) after 8-12 weeks of ICI treatment.

		Disease Control Rate				Objective Response Rate			
Variable		Univariable Analysis		Multivariable Analysis		Univariable Analysis		Multivariable Analysis	
		OR (95%CI)	*p*-value	OR (95%CI)	*p*-value	OR (95%CI)	*p*-value	OR (95%CI)	*p*-value
**Lymphocytes**	count	1.60 (0.30-8.50)	0.581	1.64 (0.22-12.30)	0.628	0.95 (0.19-4.68)	0.952	1.29 (0.21-7.84)	0.785
**B-cells – total**	count	1.75 (0.28-11.15)	0.554	1.06 (0.14-7.85)	0.951	4.67 (0.80-27.10)	0.086	5.07 (0.75-34.20)	0.095
	% lymph	0.63 (0.12-3.22)	0.574	0.28 (0.04-2.13)	0.220	1.22 (0.25-6.11)	0.807	0.91 (0.15-5.33)	0.913
**CD21- B-cells**	count	0.63 (0.12-3.22)	0.574	0.23 (0.28-1.89)	0.170	1.22 (0.25-6.11)	0.807	0.88 (0.15-5.24)	0.891
	% B	0.11 (0.02-0.72)	**0.021**	0.05 (0.00-0.67)	**0.024**	0.18 (0.03-1.09)	0.061	0.09 (0.01-0.96)	**0.046**
**Unswitched memory B-cells**	count	1.35 (0.21-8.82)	0.757	0.74 (0.09-5.76)	0.771	3.11 (0.53-18.38)	0.210	3.04 (0.45-20.37)	0.253
	% B	0.40 (0.08-2.12)	0.282	0.30 (0.04-2.04)	0.217	0.47 (0.10-2.29)	0.348	0.39 (0.07-2.10)	0.271
**Transitional Zone B-cells**	count	1.25 (0.25-6.24)	0.785	1.15 (0.16-8.11)	0.886	4.28 (0.80-22.93)	0.090	3.88 (0.61-24.74)	0.151
	% B	2.08 (0.39-11.06)	0.390	2.52 (0.39-16.29)	0.330	2.80 (0.45-17.38)	0.269	3.10 (0.45-21.21)	0.248
**Naive B-cells**	count	2.80 (0.45-17.38)	0.269	2.86 (0.40-20.79)	0.298	4.88 (0.90-26.42)	0.066	5.63 (0.93-34.05)	0.060
	% B	7.00 (1.10-44.61)	**0.039**	12.31 (1.13-134.22)	**0.039**	3.33 (0.63-17.57)	0.156	4.41 (0.70-27.74)	0.113
**Switched memory B-cells**	count	1.00 (0.18-5.46)	1.000	0.54 (0.08-3.87)	0.541	1.60 (0.31-8.25)	0.574	1.20 (0.20-7.16)	0.845
	% B	0.06 (0.01-0.62)	**0.018**	0.06 (0.01-0.70)	**0.025**	0.23 (0.04-1.25)	0.090	0.18 (0.03-1.18)	0.073
**CD24^+^CD38^++^ Regulatory B-cells**	count	NA		NA		NA		NA	
	% B	NA		NA		NA		NA	
**Plasmablasts**	count	1.00 (0.20-5.00)	1.000	0.34 (0.03-4.15)	0.395	1.33 (0.27-6.50)	0.722	0.86 (0.12-6.19)	0.878
	% B	1.00 (0.18-5.46)	1.000	0.92 (0.12-7.03)	0.938	1.27 (0.24-6.82)	0.778	0.99 (0.16-6.07)	0.989

% lymph – percent of total lymphocytes; % B – percent of total B-cells; NA – not applicable, significant values are highlighted in bold.

**Table 4:** Absolute changes of B-cells after 8-12 weeks of ICI treatment and associations with disease control rate (DCR) and objective response rate (ORR).

		Disease Control Rate				Objective Response Rate			
Variable		Univariable Analysis		Multivariable Analysis		Univariable Analysis		Multivariable Analysis	
		OR (95%CI)	*p*-value	OR (95%CI)	*p*-value	OR (95%CI)	*p*-value	OR (95%CI)	*p*-value
**Lymphocytes**	count*	1.00 (0.99-1.01)	0.630	1.00 (0.99-1.01)	0.755	1.00 (0.99-1.01)	0.748	1.00 (0.99-1.01)	0.635
**B-cells – total**	count*	0.99 (0.86-1.14)	0.907	0.96 (0.78-1.19)	0.724	0.98 (0.86-1.12)	0.776	0.96 (0.84-1.11)	0.609
	% B	1.09 (0.81-1.45)	0.577	0.98 (0.69-1.40)	0.931	1.13 (0.84-1.53)	0.416	1.07 (0.77-1.49)	0.691
**CD21- B-cells**	count**	0.99 (0.95-1.04)	0.778	0.98 (0.92-1.04)	0.514	0.99 (0.94-1.03)	0.470	0.98 (0.94-1.02)	0.340
	% B***	0.32 (0.11-0.95)	**0.040**	0.19 (0.04-0.81)	**0.025**	0.49 (0.21-1.19)	0.116	0.34 (0.12-0.93)	**0.036**
**Unswitched memory B-cells**	count**	0.96 (0.78-1.20)	0.730	0.94 (0.71-1.25)	0.663	1.00 (0.83-1.21)	0.999	0.98 (0.81-1.19)	0.822
	% B	0.95 (0.75-1-20)	0.655	0.91 (0.70-1.19)	0.494	0.99 (0.79-1.23)	0.898	0.96 (0.76-1.21)	0.709
**Transitional Zone B-cells**	count**	1.08 (0.86-1.35)	0.525	1.05 (0.80-1.37)	0.742	1.12 (0.85-1.47)	0.427	1.11 (0.83-1.50)	0.484
	% B***	2.47 (0.58-10.48)	0.220	2.27 (0.54-9.61)	0.265	3.95 (0.76-20.53)	0.102	4.38 (0.88-21.80)	0.071
**Naive B-cells**	count*	1.01 (0.84-1.21)	0.931	1.00 (0.80-1.26)	0.976	0.99 (0.82-1.18)	0.879	0.97 (0.80-1.17)	0.738
	% B***	1.54 (0.85-2.79)	0.156	1.94 (1.05-3.59)	**0.035**	1.67 (0.87-3.20)	0.122	2.15 (1.07-4.34)	**0.033**
**Switched memory B-cells**	count**	0.97 (0.87-1.09)	0.619	0.96 (0.83-1.10)	0.528	1.00 (0.91-1.10)	0.970	0.99 (0.90-1.09)	0.886
	% B***	0.30 (0.07-1.32)	0.110	0.26 (0.05-1.38)	0.113	0.81 (0.30-2.23)	0.688	0.64 (0.21-1.93)	0.426
**CD24^+^CD38^++^ Regulatory B-cells**	count	NA		NA		NA		NA	
	% B	NA		NA		NA		NA	
**Plasmablasts**	count**	1.21 (0.81-1.82)	0.349	1.08 (0.63-1.87)	0.780	1.05 (0.70-1.56)	0.827	0.94 (0.57-1.54)	0.795
	% B	1.09 (0.88-1.34)	0.446	1.09 (0.84-1.41)	0.514	0.98 (0.88-1-08)	0.622	0.97 (0.87-1.08)	0.590

% B – percent of total B-cells, *per 1000 unit, **per 100 unit, ** per 10 unit increase; NA – not applicable, significant values are highlighted in bold.

Conversely, an increase of the frequency of switched memory B-cells was significantly associated with reduced odds for DCR in the univariable (OR=0.06, 95%CI=0.01-0.62, *p*=0.018) and multivariable (OR=0.06, 95%CI=0.01-0.70, *p*=0.025) logistic regression models. When considering ORR, the results were trending towards the same direction in both, the uni- and multivariable model (**Table 3**). However, there were no significant associations with neither DCR nor ORR when considering absolute and relative changes of the frequency of switched memory B-cells (**Table 4** and **Supplementary Table 3**).

**Figure 3:**
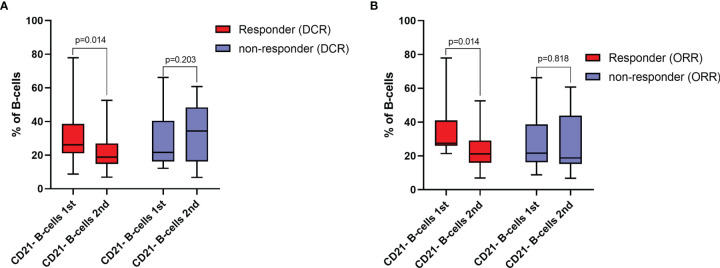
**(A)** Frequencies of CD21^-^ B-cells at baseline (1^st^ blood draw) and after 8-12 weeks (2^nd^ blood draw) of immune checkpoint inhibitor (ICI) treatment in responders and non-responders for disease control rate (DCR). Values are in percentages of total B-cells. **(B)** frequencies of CD21^-^ B-cells at baseline (1^st^ blood draw) and after 8-12 weeks (2^nd^ blood draw) of ICI treatment in responders and non-responders for objective response rate (ORR). Values are in percentages of total B-cells. All groups compared by paired Sign-Rank-Test.

Patients who had an increase in the frequency of CD21^-^ B-cells during ICI treatment were less likely to respond to ICI treatment, as indicated by DCR in univariable (OR=0.11, 95%CI=0.02-0.72, *p*=0.021) and multivariable analyses adjusted for tumor type (OR=0.05, 95%CI=0.00-0.67, *p*=0.024). Concerning ORR, an increase in the frequency of CD21^-^ B-cells was not significantly associated with response rate in the univariable model (OR=0.18, 95%CI=0.03-1.09, *p*=0.061), while it was a significant negative predictor for response when adjusted for tumor entity (OR=0.09, 95%CI=0.01-0.96, *p*=0.046) (**Table 3** and **Figure 3**). Similar results could be observed when considering absolute changes of the frequency of CD21^-^ B-cells between the first and second blood draw. An increase was significantly negatively linked to DCR in uni- (per 10-unit increase: OR=0.32, 95%CI=0.11-0.95, *p*=0.040) and multivariable analyses (per 10-unit increase: OR=0.19, 95%CI=0.04-0.81, *p*=0.025). Concerning ORR, an absolute change was not associated with ORR in the univariable analysis, but was a significant predictor of response when adjusted for tumor entity (per 10-unit increase: OR=0.34, 95%CI=0.12-0.93, *p*=0.036) (**Table 4**). A relative change was numerically linked to DCR in uni- and multivariable analyses, yet the association was not statistically significant. Considering ORR, although close to significance, a relative change in the frequency of CD21^-^ B-cells was not associated with ORR in the univariable analysis (OR=0.09, 95%CI=0.01-1.25, *p*=0.073), but reached statistical significance in the multivariable logistic regression model (OR=0.21, 95%CI=0.00-0.71.9, *p*=0.032).

## 4 Discussion

There is evidence that B-cells and TLS in the tumor microenvironment might be linked to ICI treatment response (14). However, the role of peripheral blood B-cells as potential biomarkers for treatment response has yet to be defined. Within our recent study, we prospectively evaluated the potential predictive value, as well as the changes over time of different B-cell subtypes in the peripheral blood of cancer patients undergoing ICI therapy. While we did not observe a statistically significant association of any B-cell subtype with treatment response at baseline, after 8-12 weeks of ICI treatment, we observed an increase of the frequency of CD21^-^ B-cells to be consistently associated with decreased odds for treatment response as indicated by both, DCR and ORR. Moreover, an increase of the frequency of naïve B-cells was significantly associated with increased odds for response, as indicated by DCR. Lastly, an increase in the frequency of switched memory B-cells was significantly negatively associated with treatment response indicated by DCR, whereas there was at least a numerical association with ORR.

The role of T-cells in cancer immunity and especially in the treatment with ICIs is well known (8, 9), however, although the importance of B-cells in forming anti-cancer immune reactions has been gradually revealed in recent years, a much better and detailed understanding of the role of different B-cell populations in cancer is still required (13). By their ability to present tumor antigens, to activate T-lymphocytes and a subsequential cytotoxic T-cell response, as well as to produce anti-tumor antibodies and cytokines, intra-tumoral B-cells and B-cells in regional tumor-draining lymph nodes support an effective anti-tumor immune response (13, 26, 27).

Recent evidence suggests that B-cell markers might be increased in tumors of patients who respond to ICI treatment (28). This was, for example, demonstrated by Helmink et al. (14) in small individual cohorts of melanoma- and RCC-patients, as well as in a TCGA (The Cancer Genome Atlas) RCC cohort. Additionally, tumor-infiltrating B-cells were previously linked to both longer survival and tumor stage in NSCLC (29, 30). Taken together, this suggests that the role of B-cells in cancer immunity might be independent of cancer entity, thus highlighting their importance across various cancer types. Furthermore, it does support the pan-cancer approach when evaluating the impact on peripheral blood B-cells on ICI treatment response in our present prospective study. In addition, Helmink at al (14). observed that B-cells in the tumor microenvironment are primarily localized in TLS and a higher density of B-cells and TLS was associated with response. Mass cytometry in tumor and blood samples in a small sample study (n=10) revealed that non-responders had a higher frequency of naïve B-cells. Moreover, intra-tumoral CXCR3^+^ switched memory B-cells were increased in responders vs. non-responders (14). Since we observed a decrease of the frequency of switched memory B-cells in peripheral blood to be associated with response in our cohort, we hypothesized that switched memory B-cells might be drawn to and are subsequentially enriched in the tumor microenvironment and TLS of responders during ICI treatment, thus resulting in a decrease in peripheral blood. Eventually, switched memory B-cells could differentiate into plasma cells (31) in the tumor microenvironment, thereby supporting an anti-cancer immune reaction. Interestingly, plasma cells were also enriched in responders in the study of Helmink et al. (14). Yet, further longitudinal studies are warranted to better evaluate this hypothesis.

Regarding our observation of naïve B-cells with ICI-response, the number of naïve-like B-cells was previously shown to be higher in tumor samples of patients responding to ICI therapy before treatment initiation (32). In line with these results, in the present study the increase of naïve B-cells during ICI treatment was associated with increased odds of response. Conversely, naïve B-cells have also been reported to be increased in tumors of non-responders to ICI treatment (14), thus definitive conclusions cannot be drawn and further studies are needed to clarify the role of naïve B-cells in ICI response.

Lastly, CD21^-/low^ B-cells that are linked to several autoimmune diseases, including rheumatoid arthritis, systemic lupus erythematosus, Sjogren’s syndrome or common variable immunodeficiency (15), were previously reported in a context of B-cell exhaustion and might represent an anergic B-cell population (15–19). Yet, their role in cancer and especially under ICI treatment is still undefined. To the best of our knowledge, the present study is the first report showing a consistent significant association of CD21^-^ B-cells in the blood of cancer patients with ICI treatment efficacy. Considering our results of an increased frequency of CD21^-^ B-cells being independently associated with decreased odds of response, an increase in peripheral blood CD21^-^ B-cells during ICI treatment may resemble B-cell exhaustion over the course of treatment and could thus be linked to the failure of ICI therapy.

A recent retrospective study found pretreatment peripheral B-cells to be significantly decreased in patients showing response to ICI treatment in a pan-cancer cohort including 75 patients (20), which we could not validate in our prospective study. However, both uni- and multivariable regression models were not implemented in the cited study and an analysis of different B-cell populations was not conducted, which should be mentioned as an important limitation (20).

Some limitations of our present study have to be noted. Firstly, due to the limited sample size, our study might be underpowered to detect smaller differences in the distribution and association of B-cell subtypes with the clinical endpoints. However, we did observe significant signals of CD21^-^, naïve and switched memory B-cells which presence in the tumor microenvironment has been previously linked to ICI treatment response. Thus, our data might be important for further planned larger scaled studies. Moreover, the clinical relevance of smaller changes may at least be questionable. Secondly, selection bias cannot be entirely excluded since the study was performed at a single center. Thirdly, due to the pan-cancer study design, follow-up protocols and ICI treatment dosing schemes may vary depending on cancer entity. Fourthly, PD-L1 expression status was missing for most patients since in most cases it was routinely assessed only in NSCLC. Fifth, we might have lost single plasmablasts due to our conservative CD19 gating.

In conclusion, different B-cell subtypes, notably CD21^-^, naïve and switched memory B-cells in the peripheral blood of cancer patients might represent potential novel biomarkers regarding treatment response during ICI therapy.

## Data Availability Statement

The datasets presented in this article are not readily available because The dataset for this study is not publicly available by request of the local ethic committee in order to protect the anonymity of the patients. Requests to access the datasets should be directed to martin.pichler@medunigraz.at.

## Ethics Statement

The studies involving human participants were reviewed and approved by ethics committee of the Medical University of Graz (29-593 ex 16/17). The patients/participants provided their written informed consent to participate in this study.

## Author Contributions

MP, MSt and DB contributed to conception and design of the study. SS organized the database. DB performed the statistical analysis. All authors contributed to the interpretation to the results. DB wrote the first draft of the manuscript. All authors contributed to manuscript revision, read, and approved the submitted version.

## Funding

This work was supported by the Austrian Science Fund (FWF; Hertha-Firnberg Grant T1112-B, to CK). TM was supported by Austrian Science Fund (FWF) grants P28854, I3792, DK‐MCD W1226, DOC-130; Austrian Research Promotion Agency (FFG) Grants 864690 and 870454; the Integrative Metabolism Research Center Graz; Austrian Infrastructure Program 2016/2017, the Styrian Government (Zukunftsfonds, doc.funds program), the City of Graz, and BioTechMed‐Graz (Flagship project DYNIMO).

## Conflict of Interest

The authors declare that the research was conducted in the absence of any commercial or financial relationships that could be construed as a potential conflict of interest.

## Publisher’s Note

All claims expressed in this article are solely those of the authors and do not necessarily represent those of their affiliated organizations, or those of the publisher, the editors and the reviewers. Any product that may be evaluated in this article, or claim that may be made by its manufacturer, is not guaranteed or endorsed by the publisher.

## Supplementary Material

Supplementary Figure 1 Gating strategy of B-cell subsets.

Supplementary Table 1 Antigens, clone and dye of the antibodies used.

Supplementary Table 2 Descriptive table of the study population.

Supplementary Table 3 Relative Changes per 100% increase of B-cells after 8-12 weeks of ICI treatment and associations with disease control rate (DCR) and objective response rate (ORR). % lymph – percent of total lymphocytes; % B – percent of total B-cells; NA – not applicable.
