# Supplementary figures and images for: Patterns of Peripheral Blood B-Cell Subtypes Are Associated With Treatment Response in Patients Treated With Immune Checkpoint Inhibitors: A Prospective Longitudinal Pan-Cancer Study
Source: Front Immunol. 2022 Apr 1;13:840207. doi: 10.3389/fimmu.2022.840207 (PMC9010871; doi:10.3389/fimmu.2022.840207)

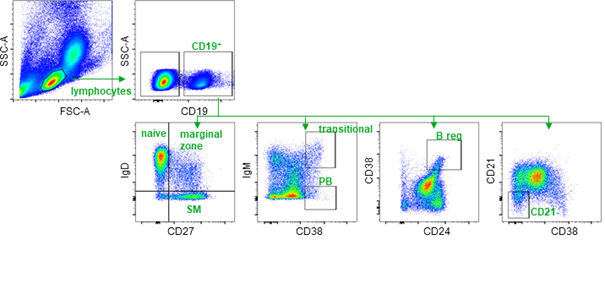

Supplement: Supplementary Figure 1 — Gating strategy of B-cell subsets. [file Image_1.png]
