# Supplementary material for: Patterns of Peripheral Blood B-Cell Subtypes Are Associated With Treatment Response in Patients Treated With Immune Checkpoint Inhibitors: A Prospective Longitudinal Pan-Cancer Study
Source: Front Immunol. 2022 Apr 1;13:840207. doi: 10.3389/fimmu.2022.840207 (PMC9010871; doi:10.3389/fimmu.2022.840207)
Supplement: Supplementary Table 1 — Antigens, clone and dye of the antibodies used. [file DataSheet_1.docx]

**Suppl. Table 3 | Antigens, clone and dye of the antibodies used**

| **antigen** | **dye** | **clone** |
| --- | --- | --- |
| CD 19 | Viogreen | LT19 |
| IgD | VioBlue | IgD26 |
| CD 24 | PerCP-Vio700 | 32D12 |
| CD 27 | APC | M-T271 |
| CD38 | FITC | IB6 |
| CD86 | PE-Vio770 | FM95 |
| CD 21 | APC-Vio770 | HB5 |
| IgM | PE | PJ2-22H3 |

**Suppl. Table 1: Descriptive table of the study population**.

|  |  | n (%miss.) | Summary measure |  |
| --- | --- | --- | --- | --- |
| **Demographic variables** |  |  |  |  |
| Sex |  | 39 (0%) |  |  |
| ---female |  |  | 12 (31%) |  |
| ---male |  |  | 27 (69%) |  |
| Age (years) |  | 39 (0%) | 64 [59-70] |  |
| BMI (kg/m²) |  | 39 (0%) | 24.4 [21.4-27.1] |  |
|  |  |  |  |  |
| **Cancer entities** |  | 39 (0%) |  |  |
| Non-small cell lung cancer |  |  | 14 (36%) |  |
| ---Adenocarcinoma |  |  | 8 |  |
| ---Squamous cell carcinoma |  |  | 5 |  |
| ---Large cell lung carcinoma |  |  | 1 |  |
| Renal Cell Carcinoma |  |  | 9 (23%) |  |
| ---clear cell |  |  | 8 |  |
| ---papillary |  |  | 1 |  |
| Head and Neck (squamous cell) |  |  | 4 (10%) |  |
| Bladder Cancer |  |  | 7 (18%) |  |
| Colorectal Cancer |  |  | 3 (7%) |  |
| Gastric Cancer (signet ring cell) |  |  | 1 (3%) |  |
| Cholangiocellular Carcinoma |  |  | 1 (3%) |  |
|  |  |  |  |  |
| History of Smoking |  | 39 (0%) | 19 (49%) |  |
|  |  |  |  |  |
| **Treatment** |  | 39 (0%) |  |  |
| ---Nivolumab |  |  | 17 (43%) |  |
| ---Nivolumab / Ipilimumab |  |  | 1 (3%) |  |
| ---Pembrolizumab |  |  | 20 (51%) |  |
| ---Atezolizumab |  |  | 1 (3%) |  |
|  |  |  |  |  |
| Treatment line |  | 39 (0%) |  |  |
| ---1^st^ line |  |  | 14 (36%) |  |
| ---2^nd^ line |  |  | 20 (51%) |  |
| ---3^rd^ line |  |  | 5 (13%) |  |

**Suppl. Table 2: Relative Changes per 100% increase of B-cells after 8-12 weeks of ICI treatment and associations with disease control rate (DCR) and objective response rate (ORR).** % lymph – percent of total lymphocytes; % B – percent of total B-cells ; NA – not applicable

|  | | **Disease Control Rate** | | | | **Objective Response Rate** | | | |
| --- | --- | --- | --- | --- | --- | --- | --- | --- | --- |
| **Variable** | | **Univariable Analysis** | | **Multivariable Analysis** | | **Univariable Analysis** | | **Multivariable Analysis** | |
|  |  | **OR (95%CI)** | ***p*-value** | **OR (95%CI)** | ***p*-value** | **OR (95%CI)** | ***p*-value** | **OR (95%CI)** | ***p*-value** |
| **Lymphocytes** | count | 2.23 (0.54-9.31) | 0.269 | 2.1 (0.43-10.34) | 0.362 | 1.24 (0.65-2.34) | 0.512 | 1.32 (0.68-2.59) | 0.414 |
| **B-cells – total** | count | 1.23 (0.60-2.49) | 0.274 | 1.12 (0.59-2.12) | 0.729 | 1.36 (0.74-2.50) | 0.351 | 1.42 (0.76-2.67) | 0.274 |
|  | % lymph | 0.74 (0.24-2.25) | 0.589 | 0.47 (0.12-1.75) | 0.257 | 1.20 (0.40-3.57) | 0.748 | 1.01 (0.30-3-38) | 0.992 |
| **CD21- B-cells** | count | 0.93 (0.49-1-76) | 0.824 | 0.82 (0.41-1.64) | 0.576 | 1.19 (0.63-2.24) | 0.589 | 1.18 (0.60-2.32) | 0.633 |
|  | % B | 0.32 (0.07-1.51) | 0.150 | 0.19 (0.03-1.33) | 0.094 | 0.09 (0.01-1.25) | 0.073 | 0.21 (0.00-0.71.9 | **0.032** |
| **Unswitched memory B-cells** | count | 1.13 (0.66-1.94) | 0.661 | 1.05 (0.63-1.75) | 0.854 | 1.24 (0.77-2.02) | 0.374 | 1.26 (0.77-2.05) | 0.335 |
|  | % B | 0.63 (0.14-2.77) | 0.537 | 0.45 (0.09-2.28) | 0.332 | 0.31 (0.05-2.07) | 0.228 | 0.21 (0.02-2.00) | 0.175 |
| **Transitional Zone B-cells** | count | 1.12 (0.89-1.40) | 0.326 | 1.10 (0.89-1.35) | 0.377 | 1.10 (0.97-1.24) | 0.154 | 1.11 (0.98-1.25) | 0.112 |
|  | % B | 1.11 (0.87-1.41) | 0.393 | 1.11 (0.87-1.43) | 0.395 | 1.20 (0.95-1.52) | 0.132 | 1.20 (0.95-1.51) | 0.123 |
| **Naive B-cells** | count | 1.38 (0.61-3.09) | 0.437 | 1.31 (0.60-2.82) | 0.498 | 1.41 (0.77-2.61) | 0.261 | 1.51 (0.79-2.89) | 0.209 |
|  | % B | 9.25 (0.22-392.46) | 0.245 | 15.04 (0.28-813.41) | 0.183 | 3.47 (0.17-70.93) | 0.418 | 7.38 (0.36-151.26) | 0.195 |
| **Switched memory B-cells** | count | 0.96 (0.56-1.66) | 0.890 | 0.90 (0.50-1.60) | 0.710 | 1.27 (0.72-2.22) | 0.412 | 1.27 (0.71-2.30) | 0.421 |
|  | % B | 0.28 (0.07-1.21) | 0.089 | 0.28 (0.06-1.32) | 0.108 | 0.29 (0.05-1.63) | 0.162 | 0.16 (0.02-1.53) | 0.112 |
| **CD24+CD38++ Regulatory B-cells** | count | NA |  | NA |  | NA |  | NA |  |
|  | % B | NA |  | NA |  | NA |  | NA |  |
| **Plasmablasts** | count | 1.09 (0.87-1.35) | 0.469 | 1.06 (0.86-1.29) | 0.600 | 1.08 (0.92-1.25) | 0.357 | 1.08 (0.92-1.27) | 0.328 |
|  | % B | 1.01 (0.81-1.26) | 0.953 | 0.96 (0.76-1.21) | 0.726 | 0.89 (0.65-1.21) | 0.448 | 0.86 (0.59-1.23) | 0.397 |
